# Differential Dose–Response Behavioural Profiles of Diazepam, Zolpidem and Indiplon in a Two-Trial Y-Maze Paradigm in Rats

**DOI:** 10.3390/ph19081282

**Published:** 2026-08-13

**Authors:** Miruna Valeria Moraru, Oana Andreia Coman, Aurelian Zugravu, Smaranda Stoleru, Cristina Isabel Viorica Ghiță, Mihnea Costescu, Elena Poenaru, Adrian-Florentin Dragomir, Aurelia Cristiana Barbu, Dragos Constantin Lunca, Andrei Stoian, Mugurel Nader Jafal, Clara Maria Stoleru, Ion Fulga

**Affiliations:** 1 Faculty of Medicine, University of Medicine and Pharmacy “Carol Davila”, 020021 Bucharest, Romania; miruna-valeria.moraru@drd.umfcd.ro (M.V.M.); oana.coman@umfcd.ro (O.A.C.); isabel.ghita@umfcd.ro (C.I.V.G.); mihnea.costescu@umfcd.ro (M.C.); elena.poenaru@umfcd.ro (E.P.); adrian-florentin.dragomir@drd.umfcd.ro (A.-F.D.); aurelia-cristiana.barbu@drd.umfcd.ro (A.C.B.); dragos.lunca@umfcd.ro (D.C.L.); andrei.stoian@umfcd.ro (A.S.); nader-mugurel.jafal@umfcd.ro (M.N.J.); ion.fulga@umfcd.ro (I.F.); 2 Faculty of Medical Engineering, National University of Science and Technology Politehnica Bucharest, 060042 Bucharest, Romania; clara.stoleru@gmail.com

**Keywords:** diazepam, zolpidem, indiplon, GABA_A_ receptors, Y-maze, exploratory behaviour, benzodiazepines, Z-drugs, behavioural pharmacology

## Abstract

**Background:** Benzodiazepines and non-benzodiazepine hypnotics (Z-drugs) act at the benzodiazepine binding site of GABA_A_ receptors but differ in their receptor subtype selectivity and behavioural profiles. Diazepam acts as a non-selective positive allosteric modulator of benzodiazepine-sensitive GABA_A_ receptors, whereas zolpidem and indiplon display preferential affinity for α1-containing receptor subtypes. Although these compounds have been investigated individually in experimental models of exploratory behaviour and cognition, comparative behavioural data obtained using the same experimental protocol remain limited. **Methods:** This study evaluated the behavioural effects of diazepam, zolpidem and indiplon in male Wistar rats using a two-trial Y-maze paradigm with a 24 h retention interval. Animals received one of three dose levels of each compound before the acquisition trial. Recall-phase behaviour was evaluated using conventional exploration measures together with time- and entry-based preference indices for the previously inaccessible arm. Primary analyses were performed separately for each compound using one-way ANOVA with appropriate post hoc comparisons. Complementary exploratory factorial analyses were subsequently conducted to examine overall behavioural patterns across drug and nominal dose categories. **Results:** Diazepam produced marked dose-dependent reductions in recall-phase exploration of the previously inaccessible arm and in both exploratory preference indices. In contrast, zolpidem and indiplon reduced exploratory activity during the acquisition phase without significantly affecting recall-phase exploratory behaviour. The complementary factorial analyses identified significant Drug × Nominal Dose Categories interactions across all recall-related endpoints, indicating that behavioural responses across the nominal dose categories differed among the three compound-specific experimental series. The strongest interaction effects were observed for Time Index % (F(6,72) = 10.522, *p* < 0.001, ηp^2^ = 0.467) and Entries Index % (F(6,72) = 6.870, *p* < 0.001, ηp^2^ = 0.364). **Conclusions:** Diazepam produced more pronounced dose-dependent alterations in recall-phase exploratory behaviour than zolpidem or indiplon in the experimental conditions employed. The complementary factorial analyses support the interpretation that the three compounds exhibited different behavioural patterns across the nominal dose categories evaluated, while these exploratory comparisons should be interpreted within the constraints of the study design. Overall, the findings contribute to the comparative behavioural characterization of benzodiazepine-site modulators in the two-trial Y-maze paradigm.

## 1. Introduction

Benzodiazepines are widely used central nervous system depressants that exert their pharmacological effects through positive allosteric modulation of the γ-aminobutyric acid type A (GABA_A_) receptor [1]. Benzodiazepines enhance GABAergic neurotransmission by binding to the benzodiazepine site of the GABA_A_ receptor complex, thereby increasing the inhibitory effects of endogenous GABA [2]. Clinically, benzodiazepines are prescribed for the management of anxiety disorders, insomnia, epilepsy, and muscle spasticity, reflecting a broad therapeutic profile [3]. Despite their clinical efficacy, benzodiazepines are associated with several adverse effects, including tolerance, dependence, and cognitive impairment [4]. One of the most clinically relevant cognitive effects is anterograde amnesia, characterized by an impaired ability to form new memories, primarily due to disruption of memory consolidation processes that transfer information from short-term to long-term storage [5,6,7]. These effects are particularly relevant in patients treated for anxiety or insomnia, populations already vulnerable to attentional and mnemonic dysfunction [8]. Consequently, benzodiazepines are recommended for short-term use and require careful dose titration and monitoring [4,9,10].

The pharmacological effects of benzodiazepines depend partly on the subunit composition of the receptor. At the molecular level, the diverse effects of benzodiazepines arise from their non-selective interaction with GABA_A_ receptors containing α1, α2, α3 and α5 subunits. Diazepam, a classical benzodiazepine, binds with high affinity and limited subunit selectivity across α1, α2, α3 and α5-containing GABA_A_ receptors [11,12].

**Figure 1:**
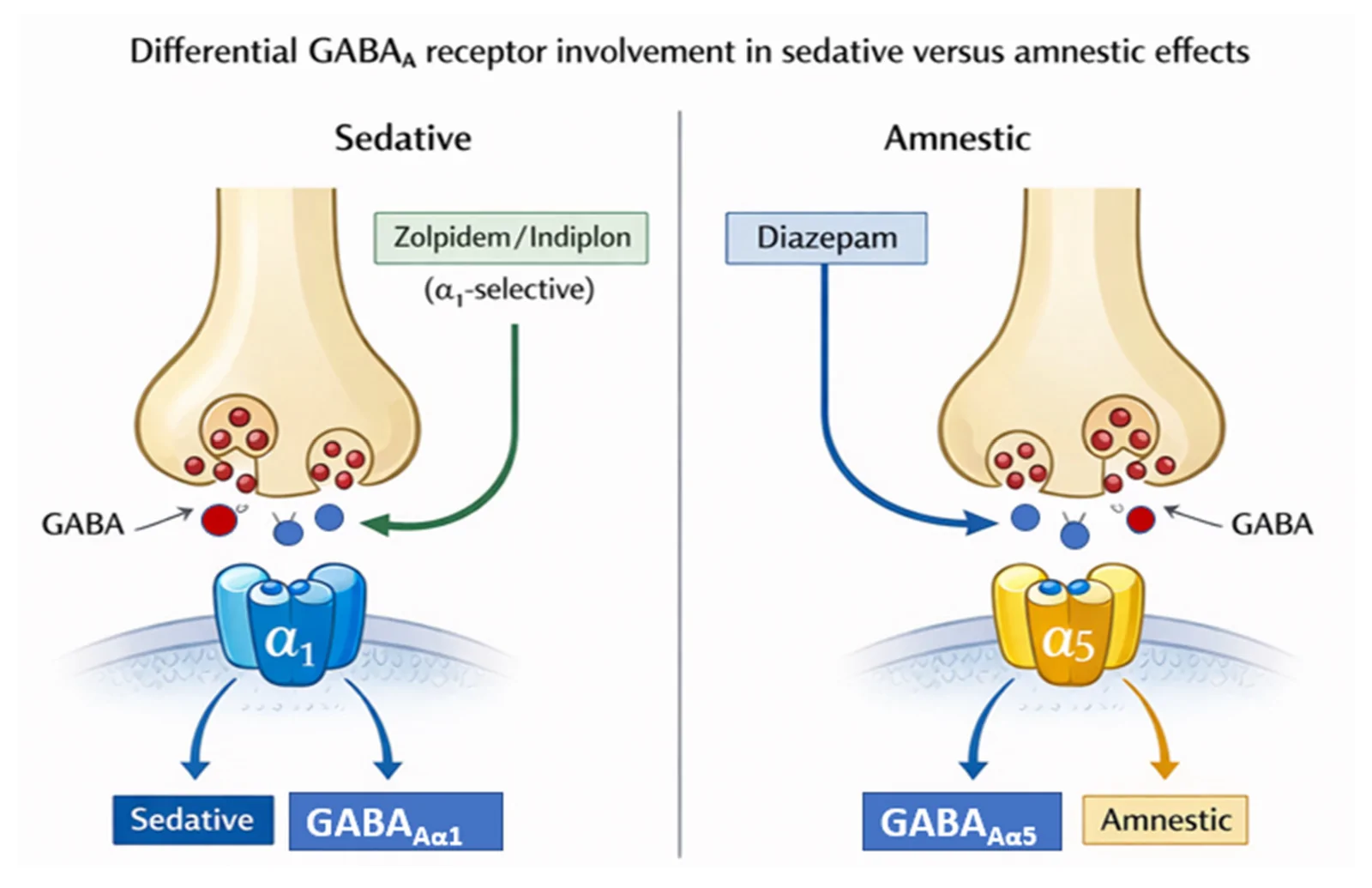
Simplified conceptual schematic illustrating previously proposed relationships between GABA_A_ receptor subtype selectivity and behavioural effects of benzodiazepine-site modulators, based on prior pharmacological literature [17,18,19,20]. The schematic is intended exclusively as a simplified conceptual representation derived from previously published pharmacological literature and does not represent a mechanistic model tested or directly demonstrated by the present experiments. The image was generated using OpenAI ChatGPT image generation. Available online: https://chatgpt.com (accessed on 20 June 2026).

Accumulating evidence suggests a functional dissociation among GABA_A_ receptor α subunits: α1-containing receptors are primarily associated with sedative and hypnotic effects, whereas α5-containing receptors, which are highly expressed in the hippocampus [13], are associated with learning and memory processes [14]. Previous pharmacological studies have suggested that α5-containing GABA_A_ receptors may contribute to some of the cognitive effects associated with benzodiazepine administration, although the precise contribution of individual receptor subtypes remains an active area of investigation [15,16]. The distinct involvement of α1- versus α5-containing GABA_A_ receptors in mediating sedative and amnestic outcomes is schematically illustrated in Figure 1.

Z-drugs were developed to treat insomnia with greater receptor selectivity and a potentially improved cognitive safety profile [21]. Compounds such as zolpidem and indiplon act at the same benzodiazepine binding site but display preferential selectivity for α1-containing GABA_A_ receptors, with relatively low affinity for α5-containing receptor subunits [21,22]. Although indiplon is not a classical Z-drug in a strict nomenclatural sense, it is frequently grouped within this category based on its non-benzodiazepine structure and predominant α1-selective pharmacological profile [22]. Zolpidem exhibits high binding affinity for α1-containing GABA_A_ receptors (pKi ≈ 7.7), approximately tenfold lower affinity for α2/α3, and negligible affinity for α5-containing receptors, whereas indiplon shows very high overall benzodiazepine-site affinity (pKi ≈ 8.8–9.0) combined with strong functional α1 selectivity and minimal activity at α5-containing receptors [22,23,24]. This pharmacological selectivity is thought to underlie their predominantly sedative–hypnotic profile and their reduced impact on memory function compared to classical benzodiazepines [25,26].

Spatial memory paradigms are commonly used to investigate hippocampus-dependent exploratory behaviour and drug-induced cognitive alterations [27,28]. The Y-maze is widely used to evaluate both acquisition and recall phases of spatial memory in rodents. A preferential exploration of a previously inaccessible arm during recall may reflect intact recognition memory and hippocampal processing, whereas a reduction or reversal of this preference may reflect altered recall-related exploratory allocation and/or altered spatial memory-related performance [28,29]. A 24 h retention interval was selected because it provides a more demanding assessment of recall-related exploratory behaviour than the shorter intervals commonly used in novelty-preference paradigms.

Although diazepam, zolpidem, and indiplon have each been investigated in experimental models of cognition and exploratory behaviour, comparative behavioural studies using the same experimental protocol remain limited. Such comparisons are relevant because diazepam acts as a non-selective positive allosteric modulator at benzodiazepine-sensitive GABA_A_ receptors, whereas zolpidem and indiplon display preferential affinity for α1-containing receptor subtypes [30,31,32]. These pharmacological differences may contribute to different behavioural patterns. However, comparisons across compounds should be interpreted with caution because the present study consisted of independent compound-specific experimental series. Accordingly, the primary objective of the present study was to characterize the behavioural effects of each compound using compound-specific one-way analyses against its corresponding vehicle control. Complementary exploratory factorial analyses were subsequently performed to examine overall behavioural patterns across the nominal dose categories investigated. Based on their different pharmacological profiles, behavioural differences among the compounds were expected. Nevertheless, the primary analyses focused on within-compound treatment effects, whereas the complementary factorial analyses were performed only as exploratory analyses to examine overall behavioural patterns across the nominal dose categories investigated.

## 2. Results

The primary analyses were first conducted separately for each compound using one-way ANOVA followed by appropriate post hoc comparisons against the corresponding vehicle control group (Dunnett’s test or Holm-adjusted pairwise comparisons, as applicable). Complementary two-way factorial ANOVA models were subsequently performed to explore overall behavioural response patterns across the nominal dose categories included in the three independent compound-specific experimental series.

### 2.1. Diazepam

During the acquisition phase (T1), diazepam did not significantly affect either exploration time or the number of entries into the open arm, although a descriptive reduction in arm entries was observed at the highest dose (Table 1).

During the recall phase (T2), diazepam produced a dose-dependent reduction in exploration of the previously inaccessible (U) arm (Table 2). One-way ANOVA showed significant treatment effects for both time spent in the U arm (F(3,24) = 29.367, *p* < 0.001, partial η^2^ = 0.786) and the number of U arm entries, as assessed using Welch’s ANOVA (F(3,12.31) = 10.235, *p* = 0.001). Compared with controls (97.60 ± 6.56 s), U arm exploration time decreased dose-dependently to 59.26 ± 6.87 s, 38.00 ± 3.41 s and 31.67 ± 4.35 s following administration of 1, 2 and 4 mg/kg diazepam, respectively.

Recall-phase exploratory preference indices were also significantly affected by diazepam treatment (Table 3). Both exploratory preference indices exceeded the theoretical chance level of 50% in vehicle-treated animals but progressively declined with increasing diazepam dose, approaching chance level at 1 mg/kg and falling below 50% at 2 and 4 mg/kg. One-way ANOVA confirmed significant treatment effects for both the Time Index (F(3,24) = 23.531, *p* < 0.001, partial η^2^ = 0.746) and the Entries Index (F(3,24) = 16.294, *p* < 0.001, partial η^2^ = 0.671). A similar dose-dependent pattern was observed for U arm entries, although significant pairwise differences were detected only at 2 and 4 mg/kg. Holm-adjusted pairwise comparisons confirmed significant reductions in both Time Index and Entries Index at all diazepam doses compared with vehicle-treated controls (Table 4).

### 2.2. Zolpidem

During the acquisition phase (T1), zolpidem did not affect time spent in the O arm, whereas the number of O arm entries decreased progressively with increasing dose (Table 5). One-way ANOVA confirmed the absence of a treatment effect on exploration time (F(3,24) = 0.194, *p* = 0.899) but identified a significant effect on the number of O arm entries (F(3,24) = 20.610, *p* < 0.001, partial η^2^ = 0.72). Dunnett-adjusted post hoc comparisons showed significantly fewer O arm entries at all zolpidem doses compared with vehicle-treated controls.

During the recall phase (T2), zolpidem did not significantly affect exploration of the previously inaccessible (U) arm (Table 6). One-way ANOVA confirmed the absence of a treatment effect on time spent in the U arm (F(3,24) = 0.398, *p* = 0.756) or on the number of U arm entries (F(3,24) = 0.286, *p* = 0.835).

Recall-phase exploratory preference indices remained above the theoretical chance level across all zolpidem doses (Table 7). No significant treatment effect was observed for the Time Index (F(3,24) = 0.121, *p* = 0.947). Although the Entries Index showed a significant overall ANOVA effect (F(3,24) = 3.184, *p* = 0.042, partial η^2^ = 0.28), Dunnett-adjusted post hoc comparisons revealed no significant differences between individual dose groups and vehicle-treated controls (Table 8).

### 2.3. Indiplon

During the acquisition phase (T1), the number of O arm entries decreased with increasing indiplon dose, whereas time spent in the O arm remained unchanged (Table 9). One-way ANOVA confirmed a significant treatment effect for O arm entries (F(3,24) = 19.471, *p* < 0.001, partial η^2^ = 0.709), whereas time spent in the O arm was not significantly affected (F(3,24) = 0.976, *p* = 0.421). The number of O arm entries decreased with increasing dose (Table 9).

Dunnett-adjusted post hoc comparisons showed significantly fewer O arm entries at 1 and 2 mg/kg compared with the corresponding vehicle control, whereas the 0.5 mg/kg dose did not differ significantly.

During the recall phase (T2), indiplon did not significantly affect exploration of the previously inaccessible (U) arm (Table 10). No significant effects were observed for time spent in the U arm (F(3,24) = 0.239, *p* = 0.868, partial η^2^ = 0.029) or for the number of U arm entries (F(3,24) = 0.343, *p* = 0.795, partial η^2^ = 0.041).

Recall-phase exploratory preference indices were not significantly affected by indiplon treatment (Table 11). Neither the Time Index (F(3,24) = 2.608, *p* = 0.075, partial η^2^ = 0.246) nor the Entries Index (F(3,24) = 1.398, *p* = 0.268, partial η^2^ = 0.149) differed significantly between groups (Table 12).

### 2.4. Complementary Factorial Analyses of Drug × Nominal Dose Category Interactions

To further explore overall behavioural response patterns, complementary two-way factorial ANOVA models were performed using drug and nominal dose category as between-subject factors. The analyses evaluated the main effects of drug and nominal dose category, together with the drug × nominal dose category interaction, for the four recall-phase behavioural endpoints.

Across all four endpoints, significant drug × nominal dose category interactions were identified, indicating that behavioural response patterns across the nominal dose categories differed among the three compound-specific experimental series. As summarized in Table 13, the largest interaction effects were observed for the Time Index (partial η^2^ = 0.467) and Time_U_T2 (partial η^2^ = 0.418), whereas smaller, but still statistically significant, interaction effects were identified for the Entries Index (partial η^2^ = 0.364) and Entries_U_T2 (partial η^2^ = 0.197).

The interaction pattern consistently indicated that behavioural responses across the nominal dose categories differed between diazepam and the two Z-drugs. Across the nominal dose categories, diazepam produced progressive reductions in recall-phase exploration and exploratory preference indices, whereas zolpidem and indiplon maintained comparatively stable behavioural responses. These findings complement the individual one-way analyses by demonstrating that behavioural response patterns differed significantly among the three compound-specific experimental series, with diazepam exhibiting a more pronounced reduction in recall-phase exploratory measures than the two Z-drugs.

**Figure 2:**
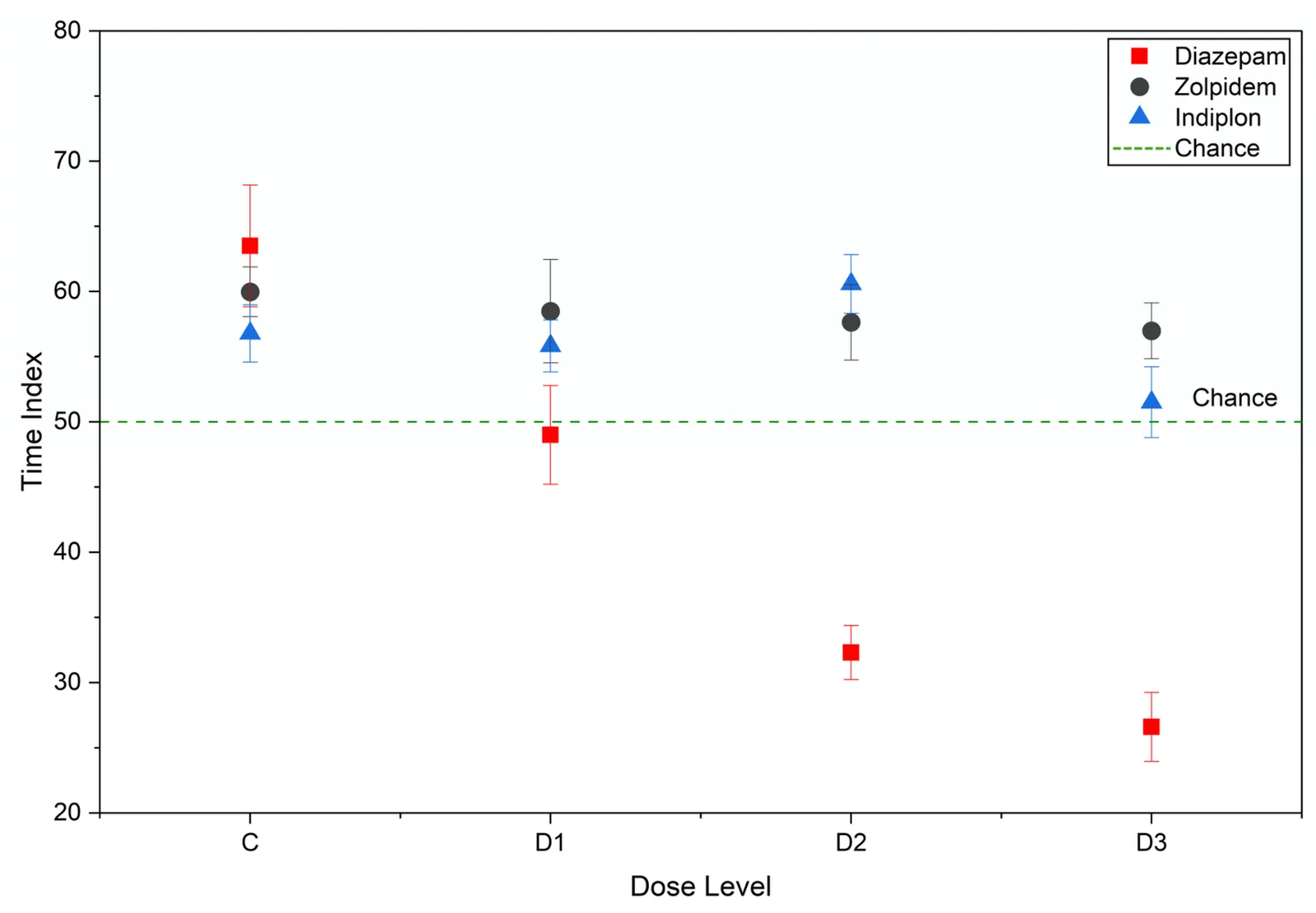
Dose-dependent effects of diazepam, zolpidem, and indiplon on recall-phase exploratory performance in the Y-maze. Time index values were calculated during recall (T2) as the percentage of exploration time spent in the unknown arm. The dashed line represents the theoretical chance level (50%). Diazepam produced a dose-dependent reduction in time index values, whereas zolpidem and indiplon produced comparatively limited changes. Values are presented as mean ± SEM for the control group (C) and dose-level groups D1–D3 (n = 7 rats/group). The significant Drug × nominal dose category interaction identified by factorial ANOVA is illustrated by the distinct behavioural pattern across the nominal dose categories observed after diazepam administration.

For the primary endpoint (Time Index %), factorial ANOVA revealed significant main effects of drug (F(2,72) = 32.562, *p* < 0.001, partial η^2^ = 0.475) and nominal dose category (F(3,72) = 13.897, *p* < 0.001, partial η^2^ = 0.367), together with a significant drug × nominal dose category interaction (F(6,72) = 10.522, *p* < 0.001, partial η^2^ = 0.467). Bonferroni-adjusted post hoc comparisons showed significantly lower Time Index values following diazepam treatment than after either zolpidem or indiplon (both *p* < 0.001). The significant interaction reflected the pronounced progressive decline across the nominal dose categories observed following diazepam administration, whereas Time Index values remained comparatively stable across the nominal dose categories for both zolpidem and indiplon. The different behavioural patterns observed across the nominal dose categories are illustrated in Figure 2.

For time spent in the unknown arm during the recall phase (Time_U_T2), significant main effects of drug (F(2,72) = 15.427, *p* < 0.001, ηp^2^ = 0.300) and nominal dose category (F(3,72) = 5.310, *p* = 0.002, ηp^2^ = 0.181) were observed. The significant drug × nominal dose category interaction (F(6,72) = 8.605, *p* < 0.001, ηp^2^ = 0.418) indicated that the behavioural changes observed across the nominal dose categories on exploration of the unknown arm differed among the three compound-specific experimental series. The descriptive pattern revealed a pronounced progressive reduction across the nominal dose categories in Time_U_T2 following diazepam administration, whereas zolpidem and indiplon maintained comparatively stable values across the nominal dose categories. A comparable pattern was observed for the exploratory preference indices.

**Figure 3:**
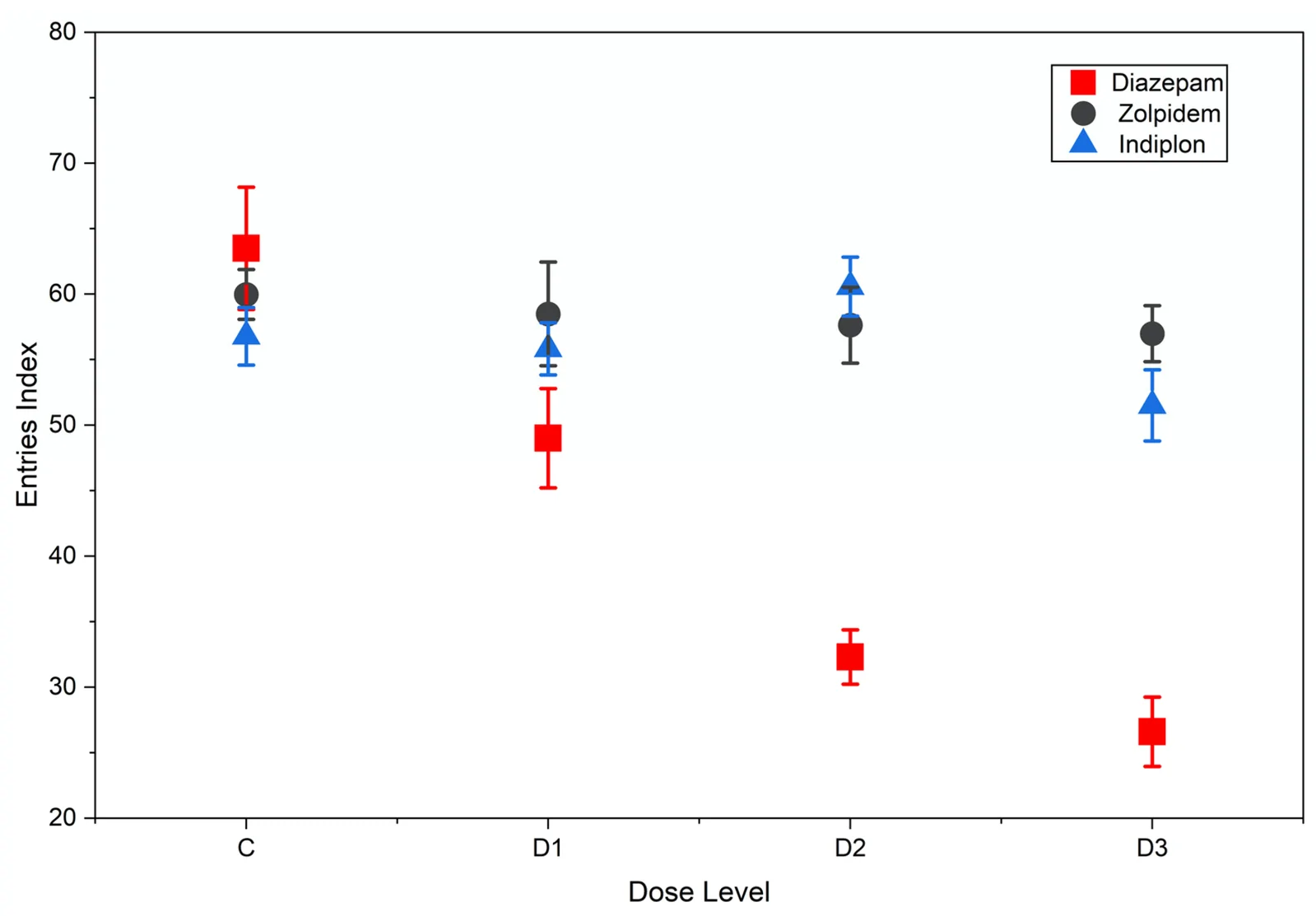
Dose-dependent effects of diazepam, zolpidem, and indiplon on Entries Index % during recall-phase Y-maze testing. Entries Index values were calculated during recall (T2) as the percentage of entries into the unknown arm relative to total entries. Values are presented as mean ± SEM for the control group (C) and dose-level groups D1–D3 (n = 7 rats/group). The significant Drug × nominal dose category interaction identified by factorial ANOVA is illustrated by the divergent behavioural pattern across the nominal dose categories observed after diazepam administration.

For Entries Index, factorial ANOVA identified significant main effects of drug (F(2,72) = 24.100, *p* < 0.001, partial η^2^ = 0.401) and nominal dose category (F(3,72) = 10.742, *p* < 0.001, partial η^2^ = 0.309), together with a significant drug × nominal dose category interaction (F(6,72) = 6.870, *p* < 0.001, partial η^2^ = 0.364). Bonferroni-adjusted comparisons showed significantly lower Entries Index values following diazepam treatment than after both zolpidem (*p* < 0.001) and indiplon (*p* < 0.001). Consistent with the Time Index results, diazepam exhibited a progressive decline across the nominal dose categories, whereas Entries Index values remained comparatively stable across the nominal dose categories for zolpidem and indiplon (Figure 3).

For the number of entries in the unknown arm during the recall phase (Entries_U_T2), the main effect of drug was significant (F(2,72) = 3.728, *p* = 0.029, partial η^2^ = 0.094), whereas the main effect of nominal dose category did not reach statistical significance (F(3,72) = 2.539, *p* = 0.063). Nevertheless, a significant drug × nominal dose category interaction was identified (F(6,72) = 2.943, *p* = 0.013, partial η^2^ = 0.197), indicating distinct behavioural patterns across the nominal dose categories among the three compound-specific experimental series. Bonferroni-adjusted comparisons showed significantly fewer entries following diazepam than zolpidem (*p* = 0.028). As observed for the other recall-phase endpoints, diazepam displayed a progressive reduction in exploration, whereas zolpidem and indiplon maintained comparatively stable behavioural profiles.

Assessment of model assumptions.

Model assumptions were evaluated using the Shapiro–Wilk test for normality of standardized residuals and the Levene test for homogeneity of variances. The results of these assumption tests are summarized in Table 14. For all four models, the Shapiro–Wilk test indicated distributions of the residuals compatible with statistical normality, with all *p*-values being above the threshold of 0.05. Similarly, the Levene test did not indicate a violation of the homogeneity of variances for any of the dependent variables.

Bonferroni-adjusted post hoc comparisons for the significant main drug effects are presented in Table 15. These comparisons summarize the overall marginal differences between the compound-specific experimental series and should be interpreted in conjunction with the factorial interaction analyses rather than as comparisons of pharmacologically equivalent doses.

## 3. Discussion

The present study compared the dose-dependent behavioural profiles of diazepam, zolpidem and indiplon using a two-trial Y-maze paradigm with a 24 h retention interval. The principal finding was that diazepam exhibited a behavioural response profile that differed substantially from those of the two Z-drugs across all recall-phase exploratory endpoints. While diazepam produced progressive reductions in exploration of the previously unknown arm and in exploratory preference indices, zolpidem and indiplon maintained comparatively stable behavioural profiles under identical experimental conditions. These findings were consistently supported by both the compound-specific one-way analyses and the complementary factorial analyses. The complementary factorial analyses further demonstrated that behavioural response patterns across the nominal dose categories differed among the three compound-specific experimental series. The consistency of these findings across both direct exploration measures and derived preference indices further supports the existence of compound-specific behavioural response profiles among benzodiazepine-site modulators [15,20,21,22]. Because the complementary factorial analyses were exploratory and based on nominal dose categories rather than pharmacologically equivalent doses, their findings should be interpreted as complementary to the primary compound-specific analyses and as supporting differences in overall behavioural response patterns rather than demonstrating comparative dose–response relationships among the tested compounds.

Behavioural studies evaluating diazepam, zolpidem, and indiplon using comparable experimental paradigms remain limited. The present study therefore extends the existing behavioural pharmacology literature by providing a standardized comparison across multiple nominal dose categories using both conventional and factorial analytical approaches.

In the Y-maze task, recall-phase behaviour is commonly evaluated using exploration-based preference indices relative to the theoretical 50% chance level. Preference values above 50% indicate preferential exploration of the previously unknown arm, whereas values near 50% suggest reduced discrimination between familiar and unfamiliar spatial contexts. Values below 50% may reflect altered recall-related exploratory allocation toward the previously explored arm [26,31].

However, because Y-maze performance may also be influenced by non-mnestic factors, including arousal, anxiety-like behaviour, or exploratory drive, the present findings should be interpreted primarily as behavioural observations obtained under the experimental conditions used in this study.

Diazepam produced a dose-dependent reduction in novelty-directed exploration during the recall phase, accompanied by progressively lower preference-index values [29]. Notably, these behavioural changes emerged at doses that did not significantly alter acquisition-phase exploratory measures, suggesting that recall-phase effects were not fully attributable to reduced exploratory activity during learning [30,31,32,33].

Although mild locomotor suppression appeared at the highest diazepam dose, changes in recall-phase exploratory behaviour were also observed at lower doses. This pattern suggests that the observed behavioural effects were not exclusively related to overt sedative activity [34,35]. Nevertheless, because independent locomotor and anxiety-related behavioural tests were not included, nonspecific influences cannot be fully excluded. The reversal of preference indices below the theoretical chance level suggests a marked alteration in recall-phase exploratory allocation.

Diazepam is a non-selective benzodiazepine-site modulator acting at GABA_A_ receptors containing α1, α2, α3 and α5 subunits [36]. Previous pharmacological studies have suggested that α5-containing receptors may contribute to some of the cognitive effects associated with benzodiazepine administration [37,38]. Consequently, the behavioural profile observed in the present study may be considered broadly compatible with previously proposed pharmacological hypotheses regarding receptor subtype selectivity. However, because the present study was based exclusively on behavioural observations, any interpretation involving specific GABA_A_ receptor subtypes should remain speculative and requires direct experimental validation. Furthermore, because complementary behavioural assays were not performed, the relative contributions of mnemonic and non-mnemonic factors cannot be definitively established [38,39,40,41].

In contrast, zolpidem and indiplon produced more limited changes in recall-phase behavioural indices across the tested dose range. Although modest reductions in exploratory activity were observed during acquisition, preference-index values during recall remained relatively stable [42,43]. These findings suggest that reduced exploratory activity during acquisition was not necessarily associated with major alterations in recall-phase novelty-directed behaviour under the present experimental conditions.

Zolpidem and indiplon are generally considered α1-preferring benzodiazepine-site modulators with relatively lower activity at α5-containing receptors [41,42]. The comparatively stable behavioural profiles observed after both Z-drugs are consistent with the relatively stable behavioural patterns observed across the nominal dose categories in the complementary factorial analyses [34,44].

Taken together, the present findings demonstrate that diazepam, zolpidem and indiplon produce clearly distinguishable behavioural response profiles when evaluated under identical experimental conditions. Rather than reflecting a uniform class effect of benzodiazepine-site modulators, the results indicate substantial compound-specific differences in recall-phase exploratory behaviour, with diazepam exhibiting the most pronounced dose-dependent behavioural alterations [45,46,47,48]. Although these findings are broadly consistent with previous pharmacological hypotheses involving differential GABA_A_ receptor subtype selectivity, the present study was based exclusively on behavioural observations and does not provide direct mechanistic evidence regarding specific receptor subtypes [49,50].

The present study also complements previous experimental work investigating pharmacologically distinct models of impaired cognitive performance, including cholinergic dysfunction and neuroinflammation. Viewed together, these experimental paradigms highlight that recall-phase exploratory behaviour in the Y-maze may be influenced through multiple neurochemical pathways, emphasizing the value of comparative behavioural pharmacology for understanding mechanism-dependent patterns of impairment [51,52].

Within the experimental conditions of the present study, these findings suggest that benzodiazepine-site modulators should not be considered behaviourally homogeneous as a pharmacological class.

### Limitations

Several limitations of the present study should be acknowledged. First, the behavioural interpretation was based exclusively on the Y-maze paradigm, which does not allow complete separation of mnemonic and non-mnemonic influences on exploratory behaviour. Although the observed recall-phase behavioural patterns are compatible with altered recall-related exploratory behaviour, factors such as arousal, anxiety-like behaviour, or exploratory drive may also have contributed to the behavioural responses observed after drug administration.

Second, independent locomotor or anxiolytic assessments were not performed. Consequently, subtle sedative, anxiolytic or motivational effects cannot be fully excluded, particularly at higher doses. While acquisition-phase exploratory activity remained relatively preserved in several groups, the absence of complementary behavioural tests limits interpretation of the specificity of the observed recall-phase effects.

Third, the study was based exclusively on behavioural observations and did not include pharmacokinetic, electrophysiological, or molecular analyses. Therefore, the proposed interpretation involving differential GABA_A_ receptor subtype selectivity should be regarded as a pharmacologically plausible framework derived from previous literature rather than as direct mechanistic evidence demonstrated by the present experiments.

Additional limitations include the relatively small sample size (n = 7/group), the exclusive use of male animals, and the absence of longer-term behavioural follow-up. In addition, although the complementary factorial analyses incorporated nominal dose categories across the three compound-specific experimental series, these categories were not intended to represent pharmacologically equivalent or equipotent doses.

Finally, although the complementary factorial analyses identified robust drug × nominal dose category interactions, the relatively small sample size per group may have limited the detection of more subtle behavioural differences between the two α1-preferring compounds.

Future studies combining complementary behavioural paradigms, locomotor and anxiety-related assessments, pharmacokinetic analyses, and receptor-specific or molecular approaches will help further clarify the mechanisms underlying the distinct behavioural response profiles observed after diazepam, zolpidem, and indiplon administration.

## 4. Materials and Methods

A total of 84 adult male Wistar rats (250–300 g) were allocated to 12 experimental groups (n = 7/group), corresponding to three independent compound-specific experimental series evaluating diazepam, zolpidem, and indiplon. Each experimental series included its own contemporaneous vehicle control group and three dose groups. Animals were housed individually under controlled temperature and humidity conditions, with a standard 12 h light/dark cycle and free access to food and water.

Animals were randomly allocated to experimental groups. Behavioural scoring was performed offline using recorded video material by an observer blinded to treatment allocation. To minimize observational bias, behavioural analysis was conducted using predefined scoring criteria applied consistently across all experimental groups. No animals were excluded from statistical analysis.

Rats were randomly assigned to diazepam, zolpidem, or indiplon treatment groups. For each compound, animals received either vehicle (control group, C) or one of three increasing dose categories (D1–D3). The selected doses of diazepam, zolpidem, and indiplon were based on previously published behavioural pharmacology studies in rodents evaluating the sedative, anxiolytic, and cognitive effects of these compounds. Diazepam doses of 1, 2 and 4 mg/kg (i.p.) were selected to explore dose-dependent behavioural effects within a pharmacological range commonly used in experimental models [42,43].

All substances were administered intraperitoneally at a final injection volume of 1 mL/kg. Control group animals received saline solution (0.9% NaCl). Diazepam was administered at doses of 1, 2 and 4 mg/kg, whereas zolpidem and indiplon were administered at doses of 0.5, 1 and 2 mg/kg.

For zolpidem and indiplon, lower dose ranges were chosen based on studies suggesting relatively limited sedative or locomotor impairment at these concentrations [44,45]. Previous experimental data indicated that indiplon doses below approximately 2.5–3 mg/kg were not consistently associated with major locomotor or vigilance impairment in rodents [44], while zolpidem doses around 3 mg/kg have been used in behavioural paradigms without marked sedative suppression [45].

The selected doses were intended to characterize within-drug behavioural profiles rather than to establish pharmacological equivalence or dose equivalence across compounds.

The 2 h interval between drug administration and behavioural acquisition was selected based on previously published experimental protocols investigating behavioural effects after systemic administration in rats and was intended to allow adequate systemic absorption and central distribution before testing [46,47,48].

The Y-maze paradigm was selected because it provides a relatively rapid and low-stress assessment of novelty-directed exploratory behaviour and recall-related exploratory performance in rodents. Compared with more demanding behavioural paradigms, such as the Morris water maze, the Y-maze requires minimal training and avoids swimming-related stress, which could potentially influence exploratory and behavioural responses after administration of sedative-hypnotic compounds. In addition, the two-phase Y-maze protocol allows evaluation of recall-phase exploratory allocation toward a previously inaccessible arm under relatively simple experimental conditions [49,50].

Behavioural assessment was performed using a two-trial Y-maze paradigm. Drugs were administered intraperitoneally (T0) 2 h before the acquisition session. During acquisition (T1), one arm of the maze remained closed, allowing exploration of the start (S) arm and one open/familiar arm (O). The recall session (T2) was conducted 24 h later, when all maze arms were accessible, allowing exploration of both the previously familiar arm (K) and the previously inaccessible unknown arm (U) [24,53,54,55,56]. A 24 h retention interval was selected to provide a more demanding assessment of recall-phase exploratory behaviour than the shorter intervals frequently used in novelty-preference paradigms.

Behavioural data are presented as mean ± standard error of the mean (SEM). The primary statistical analyses were performed separately for each compound (diazepam, zolpidem, and indiplon) using one-way analysis of variance (ANOVA), followed by Dunnett’s multiple-comparison test to compare each dose group with its corresponding vehicle control. For the statistical analysis was used IBM SPSS Statistics for Windows, version 29.0 (IBM Corp., Armonk, NY, USA).

This analytical strategy reflected the experimental design, in which each compound was evaluated in an independent compound-specific experimental series with its own vehicle-treated control group. Consequently, the primary statistical inference was based on within-compound comparisons rather than direct comparisons across compounds.

To further explore whether behavioural response patterns differed among compounds, complementary factorial two-way ANOVA models were performed using Drug and nominal dose categories (D1–D3) as between-subject factors. In these complementary statistical analyses, the nominal dose category represented the lowest, intermediate, and highest dose tested for each compound rather than equivalent absolute doses across drugs. Therefore, these complementary factorial analyses were not intended to compare pharmacologically equivalent doses or to establish comparative dose–response relationships among compounds, but rather to examine whether overall behavioural response patterns differed across the nominal dose categories investigated.

Four dependent variables were analyzed: Time Index (%), Time_U_T2, Entries Index (%), and Entries_U_T2. Main effects of Drug, nominal dose category, and Drug × nominal dose category interactions were evaluated. For the complementary factorial analyses, Bonferroni-adjusted post hoc comparisons were performed when significant overall effects or interactions were observed. The factorial analyses should therefore be interpreted as exploratory and complementary to the primary compound-specific analyses.

Model assumptions were assessed using the Shapiro–Wilk test for normality of residuals and Levene’s test for homogeneity of variances. Effect sizes were expressed as partial eta-squared (ηp^2^), together with 95% confidence intervals where available. Statistical significance was defined as *p* < 0.05.

## 5. Conclusions

The present study demonstrated that diazepam, zolpidem, and indiplon exhibited distinct behavioural response patterns when evaluated under identical experimental conditions in a two-trial Y-maze paradigm. Within the individual compound-specific analyses, diazepam produced progressive dose-dependent reductions in recall-phase novelty-directed exploration and exploratory preference indices, whereas the two α1-preferring Z-drugs maintained comparatively stable behavioural profiles across their respective dose ranges.

The consistency of these findings across direct exploration measures, derived preference indices, and complementary factorial analyses supports the existence of compound-specific behavioural response patterns among benzodiazepine-site modulators. The complementary factorial analyses should be interpreted as reinforcing the primary compound-specific findings by demonstrating differences in overall behavioural response patterns across the nominal dose categories investigated.

Although the present findings are based exclusively on behavioural observations and do not provide direct mechanistic evidence regarding GABA_A_ receptor subtype involvement, they establish a standardized behavioural framework for future comparative investigations of benzodiazepine-site modulators and other subtype-selective GABA_A_ receptor ligands.

## Figures and Tables

**Table 1:** Effects of Diazepam on Exploration During Acquisition Phase (T1).

Diazepam Dose (mg/kg)	Time in O Arm T1 (s, Mean ± SEM)	Entries in O Arm T1 (Mean ± SEM)
0 (Control)	89.23 ± 4.30	6.29 ± 0.52
1	93.29 ± 5.59	5.29 ± 0.52
2	79.87 ± 5.29	5.14 ± 0.55
4	101.13 ± 15.60	4.29 ± 0.47

**Table 2:** Effects of Diazepam on Exploration During Recall Phase (T2).

Diazepam Dose (mg/kg)	Time in U Arm T2 (s, Mean ± SEM)	Entries in U Arm T2 (Mean ± SEM)
0 (Control)	97.60 ± 6.56	6.57 ± 0.65
1	59.26 ± 6.87	4.86 ± 0.55
2	38.00 ± 3.41	3.14 ± 0.51
4	31.67 ± 4.35	3.00 ± 0.22

**Table 3:** Effects of Diazepam on Exploratory Preference Indices During Recall Phase (T2).

Diazepam Dose (mg/kg)	Time Index % * (Mean ± SEM)	Entries Index % (Mean ± SEM)
0 (Control)	63.5 ± 4.67	59.1 ± 4.11
1	49.0 ± 3.79	48.1 ± 2.72
2	32.3 ± 2.08	35.6 ± 1.83
4	26.6 ± 2.64	36.4 ± 1.67

* The time index represents the percentage of total recall-phase exploration allocated to the previously inaccessible (U) arm and was calculated as: (time spent in the U arm/total time spent in both arms) × 100.

**Table 4:** Summary of one-way ANOVA results for diazepam.

Dependent Variable	F-Statistic	*p*-Value	ηp^2^
Entries in O Arm T1	2.500	0.160	0.238
Time in O Arm T1	0.962	0.230	0.107
Time in U arm T2	29.367	<0.001	0.786
Entries in U arm T2	10.235 (3; 12.31), Welch	0.001	-
Time index (%)	23.531	<0.001	0.746
Entries index (%)	16.294	<0.001	0.671

**Table 5:** Effects of Zolpidem on Exploration During Acquisition Phase (T1).

Zolpidem Dose (mg/kg)	Time in O Arm T1 (s, Mean ± SEM)	Entries in O Arm T1 (Mean ± SEM)
0 (Control)	99.33 ± 7.03	5.57 ± 0.43
0.5	94.57 ± 14.22	4.14 ± 0.26
1	82.61 ± 15.05	3.00 ± 0.38
2	92.24 ± 23.23	1.71 ± 0.36

**Table 6:** Effects of Zolpidem on Exploration During Recall Phase (T2).

Zolpidem Dose (mg/kg)	Time in U Arm T2 (s, Mean ± SEM)	Entries in U Arm T2 (Mean ± SEM)
0 (Control)	69.7 ± 4.91	5.43 ± 0.61
0.5	79.6 ± 7.76	6.00 ± 0.53
1	78.2 ± 8.05	5.29 ± 0.52
2	76.0 ± 6.74	5.43 ± 0.69

**Table 7:** Exploratory Preference Indices During Recall Phase (T2) for Zolpidem.

Zolpidem Dose (mg/kg)	Time Index (%) Mean ± SEM	Entries Index (%) Mean ± SEM
0 (Control)	59.98 ± 1.90	59.29 ± 1.67
0.5	58.49 ± 3.96	61.97 ± 3.00
1	57.63 ± 2.90	52.21 ± 2.79
2	56.98 ± 2.14	54.92 ± 1.57

**Table 8:** Summary of one-way ANOVA results for zolpidem.

Dependent Variable	F-Statistic	*p*-Value	ηp^2^
Entries in O Arm T1	20.610	<0.001	0.720
Time in O Arm T1	0.194	0.899	0.024
Time in U Arm T2	0.398	0.756	0.047
Entries in U Arm T2	0.286	0.835	0.035
Time Index	0.121	0.947	0.015
Entries Index	3.184	0.042	0.285

**Table 9:** Effects of Indiplon on Exploration During Acquisition Phase (T1).

Indiplon Dose (mg/kg)	Time in O Arm T1 (s, Mean ± SEM)	Entries in O Arm T1 (Mean ± SEM)
0 (Control)	110.60 ± 8.52	6.00 ± 0.38
0.5	98.03 ± 7.37	5.29 ± 0.68
1	111.40 ± 31.91	2.57 ± 0.37
2	65.94 ± 27.71	1.57 ± 0.43

**Table 10:** Effects of Indiplon on Exploration During Recall Phase (T2).

Indiplon Dose (mg/kg)	Time in U Arm T2 (s, Mean ± SEM)	Entries in U Arm T2 (Mean ± SEM)
0 (Control)	76.70 ± 7.92	4.86 ± 0.86
0.5	82.27 ± 5.10	5.29 ± 0.94
1	83.43 ± 7.40	4.86 ± 0.34
2	78.20 ± 5.41	5.71 ± 0.47

**Table 11:** Exploratory Preference Indices During Recall Phase (T2).

Indiplon Dose (mg/kg)	Time Index (%) Mean ± SEM	Entries Index (%) Mean ± SEM
0 (Control)	56.78 ± 2.19	49.11 ± 2.27
0.5	55.82 ± 1.99	55.31 ± 2.34
1	60.58 ± 2.25	53.30 ± 2.73
2	51.50 ± 2.72	50.88 ± 1.52

**Table 12:** Summary of one-way ANOVA results for indiplon.

Dependent Variable	F-Statistic	*p*-Value	ηp^2^
Entries in O Arm T1	19.471	<0.001	0.709
Time in O Arm T1	0.976	0.421	0.109
Time in U Arm T2	0.239	0.868	0.029
Entries in U Arm T2	0.343	0.795	0.041
Time Index	2.608	0.075	0.246
Entries Index	1.398	0.268	0.149

**Table 13:** Summary of two-way factorial ANOVA results for the four recall-phase behavioural endpoints.

Dependent Variable	Drug Effect	ηp^2^	Nominal Dose Category Effect	ηp^2^	Drug × Nominal Dose Category Interaction	ηp^2^	R^2^ Adjusted
Time Index %	F(2,72) = 32.562; *p* < 0.001	0.475	F(3,72) = 13.897; *p* < 0.001	0.367	F(6,72) = 10.522; *p* < 0.001	0.467	0.657
Time_U_T2	F(2,72) = 15.427; *p* < 0.001	0.300	F(3,72) = 5.310; *p* = 0.002	0.181	F(6,72) = 8.605; *p* < 0.001	0.418	0.513
Entries Index %	F(2,72) = 24.100; *p* < 0.001	0.401	F(3,72) = 10.742; *p* < 0.001	0.309	F(6,72) = 6.870; *p* < 0.001	0.364	0.571
Entries_U_T2	F(2,72) = 3.728; *p* = 0.029	0.094	F(3,72) = 2.539; *p* = 0.063	0.096	F(6,72) = 2.943; *p* = 0.013	0.197	0.207

**Table 14:** Assessment of model assumptions: normality of residuals and homogeneity of variances.

Dependent Variable	Shapiro–Wilk for Residuals	Statistical Normality	Levene Test	Homogeneity
Time Index %	W = 0.991; *p* = 0.832	ok	F(11,72) = 1.170; *p* = 0.323	ok
Time_U_T2	W = 0.986; *p* = 0.507	ok	F(11,72) = 0.745; *p* = 0.692	ok
Entries Index %	W = 0.984; *p* = 0.389	ok	F(11,72) = 1.253; *p* = 0.270	ok
Entries_U_T2	W = 0.981; *p* = 0.257	ok	F(11,72) = 1.294; *p* = 0.246	ok

**Table 15:** Bonferroni-adjusted post hoc comparisons for significant main drug effects.

Variable	Bonferroni Comparison	Mean Difference	*p* Adjusted	Interpretation
Time Index %	Diazepam vs. Zolpidem	−15.223	<0.001	Diazepam associated with lower values
Time Index %	Diazepam vs. Indiplon	−13.227	<0.001	Diazepam associated with lower values
Time_U_T2	Diazepam vs. Zolpidem	−19.229	<0.001	Diazepam associated with lower values
Time_U_T2	Diazepam vs. Indiplon	−23.511	<0.001	Diazepam associated with lower values
Entries Index %	Diazepam vs. Zolpidem	−11.990	<0.001	Diazepam associated with lower values
Entries Index %	Diazepam vs. Indiplon	−7.406	<0.001	Diazepam associated with lower values
Entries Index %	Zolpidem vs. Indiplon	4.584	0.031	Zolpidem > indiplon
Entries_U_T2	Diazepam vs. Zolpidem	−1.143	0.028	Diazepam associated with lower values

## Data Availability

The original contributions presented in this study are included in the article. Further inquiries can be directed to the corresponding authors.
